# Automated systems for the clinical laboratory: the user's needs

**DOI:** 10.1155/S1463924680000235

**Published:** 1980

**Authors:** J. Bierens de Haan

**Affiliations:** Laboratoire Riotton sa 16. bd des Tranchees Geneva CH11206 Switzerland


### Automated systems for the clinical laboratory: the user's needs

**J. Bierens de Haan**
*Laboratoire Riotton sa, 16. bd des Tranchees, CH1206 Geneva, Switzerland*

### Cooperation

Clinical chemists 'discovered' automation some twenty years ago and, as instrument users, have spent these last two decades alternating between hope and deception. They are now disillusioned people. Each year brings a fresh crop of original systems, but chemists have found that they have only one chance in ten of purchasing a useful instrument since there is no way of telling the good from the bad on an exhibition stand.

I remember a closed meeting held in Munich in 1978 between users and suppliers of laboratory products, which discussed the recurring problems of communication and cooperation. A top executive of a leading company, apparently exasperated by the usual mistrusting comments of the laboratory professionals, suddenly burst out 'The instrument companies have raised your profession to its present status. Without us you would still be playing with test tubes and bunsen burners down in the basements of your hospitals! You can't just go on ignoring what you owe to industry, treating us as junior partners, closing the door in our faces when we try to join your panels but at the same time asking for more and more free reagent samples and unlimited, unconditional instrument evaluation. . . .' This is the gist of what he said, but he was most sincerely upset by the reaction of the laboratory professionals.

Meanwhile, I was wondering why it was that an instrument evaluation in perhaps another country or by a different laboratory is never good enough for one's own purposes. Instrument evaluation is a highly expensive activity in our laboratories even without such duplication. I realized then that I wasn't the only one to be unhappy with the situation, and that the communication gap was probably much larger than I had suspected. No matter how much closer to each other the two sides may draw, I cannot see the clinical chemist feeling indebted to industry for having contributed to the present status of his profession, nor will he expect any acknowledgement from industry. Cooperation in this context simply means that we try to add rather than cancel our respective efforts and expertise in an area of common interest. It is a means to self-realization, and has not a great deal to do with mutual support.

Indeed, we can only agree on means: our aims are different. Industry sells, we analyse. The common area is the instrument, but its definition is equivocal: for industry it is a product, to us it is a tool. And all too often it is prototype - and a nuisance. Good communication must be sought at a still more basic level. Only when we have agreed proper definitions for such terms as 'sample throughput', 'throughput time', 'volume ratio', 'photometric accuracy', 'carry-over' etc., will those terms carry unequivocal information.

The role of industry as a leading force in the technological development of laboratories cannot be questioned. What can be questioned is the ultimate value of this development for which industry cannot be completely blameless. The truth is that we have no reason to be too proud of clinical chemical practice today. But if industry insists on sharing credit for our successes, it must also be prepared to accept some responsibility for our failures.

It is not my intention here to detail the successes, since they have been exhaustively discussed elsewhere [1]. It is undeniable that the remarkable improvements in kinetic analysis or particle counting, for example, owe a great deal to new technologies and demonstrate that cooperation can be fruitful. For the purpose of this paper I would like to elaborate on the three points introduced above: the unhappiness, the communication gap, and the degradation of clinical laboratory practice. I will then conclude with the user's needs.

As a preface, let me make one general statement: I am neither complaining, nor casting stones. I am trying to understand. People are no better and no worse whether they belong to industry, to scholarship, or to medicine. However, the systems, i.e. the existing relationship between each decision group, must be criticized. This is a prerequisite for positive change, which in turn can only come from individual initiatives.

### Unhappiness

I have been but a moderately satisfied user of automated instruments for more than ten years (automated or mechanized - let us not play with definitions for once: everything beyond the Dubosq colorimeter can be called automated). My experience is that high-capacity instruments do not completely solve our problems. The manpower costs we save on performing analyses will be required for evaluating, calibrating, and maintaining the instruments. Even when the instruments are of clever design, they are often unreliable in performance and construction; the run-in time is too long, often over six months; the training of operators non-existent; the breakdowns too frequent; the service poor. Powerful instruments can demand that the laboratory is re-organized around them with little obvious advantage. As such they disorganise the existing structure and make themselves even more unrewarding for the staff. They build up bottle-necks because speed is often sacrified for automation. Details of such dissatisfaction are available elsewhere. Every statement in the recently issued IUPAC document on the subject [2], addresses a problem encountered in present day instruments. Some statements are almost funny, like that on interaction within the instrument. This could better read 'an instrument should not destroy itself'.

### Communication?

I thus have unsatisfied needs which I believe I can describe in detail and for which technologically and economically sound solutions must exist. However, I am not sure that I can convince anybody. In fact, I don't think manufacturers are really interested.

Let us consider the Du Pont 'aca', for example. To me it is a black box which surrenders the clinical chemist, handcuffed, to one company. My definition of a black box here is an instrument with an input and an output and something in between which you are not really interested in because you cannot do anything about it when it fails. Even calibration and quality control are biased concepts with this machine. I am an analyst, and look at a clinical laboratory as a living entity with tests, methods, and instruments that are born, live, and die, with an average life, and a thrilling one, of perhaps five to seven years. An 'aca' would make my laboratory sick. But what is the point of claiming that the instrument is ill-conceived when it was obviously well enough conceived to allow the manufacturers to sell over a thousand (approximately 1800 sold up to July 1979) units around the world, some to quite prestigious laboratories? The instrument entered the world market as a routine instrument and not just as a prototype, and it was among the very few that stood up to the harsh demands of the laboratory. It was thus selected by our institutions as one of the more useful instruments, if not the best in scientific terms. The struggle for survival involves characteristics other than those of pure analysis, such as ease of use, ruggedness, training and servicing, updating of application, etc. The 'aca' is a success. It is simply a matter of viewpoint. If there is a market for such 'package-processors' or if, more generally, a sufficient proportion of users do not object to being forced to buy their reagents and methods from the instrument manufacturer, then merger and consolidated policy will continue until the trend towards such systems becomes irreversible whatever the consequence to our freedom of choice and ultimately to health care.

Industry, by its very nature, looks for markets; the larger the return and the shorter the term the better. It may be that the need I think I have identified represents such a market. But it is more likely that a slightly different need will represent a better market. So the manufacturer will shift his target and try to persuade me that he knows my need better than I do. Sometimes this is done quite sincerely. For example, laboratory people originally wanted a facility for specimen-splitting in order to distribute labelled material to the bench, together with the work sheet, and so retain the measuring instrument of their choice at each working station. They are still waiting. Some limited attempts were made in the late 1960's. One by Beckman was stillborn. Another by Siemens was dropped only recently after some ten years of painful prototyping. Today's solution, if the project were to be restarted from zero, might be extremely elegant and so-called 'limited automation' would do the rest. However, by now most of us have given up the idea and have learnt to accept those monstrous multichannel instruments nobody ever asked for. And we have even started to believe that we needed this sort of equipment to solve our internal organization problems. One tube - twelve or twenty results: a dream! Or a nightmare when it comes to the inevitable analytical compromises, the failure rate and restart problems, the lack of flexibility, the staff-rejection syndrome etc. The actual efficiency of high-capacity analyzers is grossly overestimated. Very often they require more time for start-up and shut-down than for analyses. As a rule, one measurement out of three or four is for calibration, control, or re-checking. These systems are praised for their administrative qualities but they are directly responsible for our administrative problems. It is a vicious circle.

The manufacturer seldom seems aware of the mismatch. He has learnt to deal with his customer as somebody who expects results on blood tests. He comes with simple ideas such as that any test can be performed if you can 1) dilute a sample; 2) add one or two reagents; 3) wait for some time; 4) read an absorbance. Or: a test result is either normal or abnormal; normal means healthy, abnormal means sick! He has only a superficial idea of his interlocutor's real concerns. In particular, he seems to ignore the permanent stress (or distress) that can result from an unreliable instrument. His own motivation is simple: he wants to sell. The clinical chemist's motivation is complex. His ambitions usually go beyond the mere output of results. He is concerned with how they are obtained and what to use them for. His academic training leaves him ill-prepared to run the industry-like production systems which our laboratories tend to become and which manufacturers or their representatives think they understand. And this obscures his critical sense. It is also true that some of his demands are extravagant and that he has little idea of their implicit costs. The management of our laboratories is not an easy task, and the manufacturer can only come up with very partial solutions to the problems.

Communication problems of a similar nature can sometimes be found inside the instrument company itself, between the more user-oriented R & D or applications people on the one hand, and an over-zealous marketing department on the other. A number of gross failures can be traced back to the latter releasing immature and therefore unviable instruments against the advice of the former.

But let me now depart from this basic criticism and assume automated instruments that perform reliably and sell. Should not these prove that demand has been fulfilled? Not really, because they never coincide with the average user's need. And this is because there is no average user. There are only users, and here manufacturers deserve credit for finding their way through such a diversity of behaviours and mentalities.

### Laboratory practice

Modern health care systems have become large consumers (one might even suggest wasters) of laboratory data. The trend, though slowing down, has not yet reversed. In France, for example, the cost of analyses shows an increase of 22% in 1978 i.e. larger than that (18.5%) of total medical consumption (price and volume contributed to these figures in a ratio of 1 to 1). Mass producing clinical chemistry is young and unsettled. Whether justified or biased, markets for instruments and reagents have expanded at a very unusual rate during the past 25 years, producing new demands, not always unselfish, among the professional and economic sectors concerned and provoking some dramatic financial failures. People producing laboratory data call themselves clinical biochemists, medical biologists, laboratory doctors, chemical pathologists, or even biological pharmacists, what they are called depends upon their geographical location as much as anything else. To be complete, one should include the very large group of medical technicians often working quite independently under the formal responsibility of a physician and last but not insignificant, the group of devoted doctors' wives who often fulfil this function. All these people operate in a widely variable and sometimes unstable cultural, economic, technical, and legal environment. Their training, if any, usually includes both medical and chemical curricula, but again in a highly variable proportion.

Within almost every country a more or less active competition exists between doctors and chemists for the control of laboratories. This is more of a stimulus in those countries where regulations have clearly delineated the rights and duties of each partner group; in France, for instance, where prescription and invoicing of tests cannot be carried out by the same agency. It is a source of considerable qualitative and quantitative bias in the instrument and reagent markets of countries where self-requesting prevails among the producers of laboratory results. In Switzerland about 50% of total medical consumption occurs outside the hospital system (500 hospitals), 5% of which, i.e. 10% of direct medical costs, are for analyses but only 1% goes through an approved laboratory. The remaining 9% (around 500 million SF) is directly charged by doctors performing tests in their own laboratory. In Germany over 65% of total medical consumption is extra-hospital (this and all subsequent figures are for 1976). Laboratory costs represent about 5% of this. They are charged by 400 private laboratories (working partly for hospitals too) and 25,000 private practitioners. The latter perform 20% of all clinical chemical tests in the country, creating an unusually large market for small kits and so-called monotests, with the corresponding increase in price and quality control problems - or, more likely just quality problems. They also perform 60% of all urine-screening, doubling the number of such strip tests per patient compared to France. Since these kits are twice as expensive, the German-to-French ratio for this specific market is five to one, accounting for the difference in the size of population.

In such a situation control of the choice of tests, amount ordered, quality of result, and profit is entirely shared between the doctor and the manufacturer. The latter, being the more competent and better organized of both partners, is in a very strong position to advise his customer, particularly about methods and instruments. Until paying third-parties and/or patients become aware of the situation, willing to question the clinical justification of the request and sufficiently organized to do so, the trend cannot be expected to reverse. And the clinical chemist can have no influence in this case as he is completely outside the flowchart. There are cases, however where perhaps he could exert influence such as in the French hospitals where 32% of all chemical tests are five electrolytes (Na, K, CI, Ca, P). An extraordinary figure, which it is tempting to link with the undoubted success of Technicon's SMA in that country.

Whether they are created by economic or legal factors and maintained by profit groups, or clinically and scientifically justified, there are considerable quantitative and qualitative differences between the two major European countries in their approach to analyses. The Germans spend exactly twice as much as the French per inhabitant for analyses, doing 6 tests where the French do 3.8. The additional 2.2 are mainly glucose and enzymes (the Germans do twice as many glucose tests per inhabitant as the French). The costs of instruments and reagents, however, are about the same in both countries. France is proportionately more automated than Germany. The Germans seem to ignore the use of calcium and potassium measurements. They do much more coagulation testing because they use prothrombin time as a hepatic parameter. Because of legal hindrance the French do four times fewer RIA's etc. Most of these figures come from a recent work by Schoff [3], which casts a searching light on our profession. Obtaining similar data for other European countries would not only show the priorities for standardization, but would also teach us humility: there are also communication gaps within the profession. But there are common features also: screening, profiling, defensive medicine, and short term profit combine to generate a systematic over-production and under-utilization of data. And industry shares the responsibility for this.

Let me complete this rather chaotic picture by insisting that the average quality of our results is still far from satisfactory and homogeneous, however tired we may be of harking back to the same old story. Automated instruments enable us to measure more specimens for more parameters but they are given undeserved credit for improving the quality of test results. Quality surveys constantly show that long term precision and accuracy of manual methods are just as good or even better. It is the quality of instruments and the value of calibrators, not the degree of mechanization, that have a decisive influence on the quality of results.

### User's needs

Looking for the user in order to identify his needs, I have been obliged to recognize a dramatic lack of coherence and efficiency in our profession. There is a danger that, having failed so far to present an organized front, clinical chemistry will be degraded into a number of marketing outlets and eventually retrieved by industry with the tacit approval of government, just like another pharmaceutical business. As everyone knows, drug inflation is considerably worse and less justified than test inflation and there is hardly any intermediary between the doctor and the manufacturer. Pharmaceutical companies are in a position where they can consciously disinform the physician, for example dissuading him from controlling his patients's drug levels, such as digoxin. Control of drug level would soon demonstrate the gross overdosage of these chemicals in the population and consequently compromise sales.

It is now a race against time. The twin origins of clinical chemistry make both for its wealth and its weakness. Each of us inevitably leans more either to the chemical or to the clinical side, so that each of us has his Achilles' heel. Industry, logically, has taken advantage of this duality from the very beginning, forcing oversimplified analytical concepts into the clinically-minded, and rudiments of diagnostics into the analytically minded. This is how such odd instruments as the Auto Analyzer were so widely accepted and how such non-tests as the CEA (carcino-embryonic antigen) test manage to enter the market. We must not allow industry to simplify our investigational methods into a caricature and immobilize our profession to a sterile infantilism.

Our laboratories and, more specifically, our instruments, are components of a formidable venture called health care. Health care, Ralph Nader has said is a cost-plus, increasingly no-fault industry with no incentive to reduce costs. And he might have added 'or to increase efficiency'. Unlike other businesses, our laboratories are production systems with no feedback be it from the doctor, the patient, or anyone else. This endows us with a considerable responsibility if we believe that clinical chemistry can contribute to improving people's living. We are alone and free to decide what we are going to demand, to accept, and to refuse. I am convinced that we could have avoided the dinosaurs.

I am not sure that industry should have concerned itself with the diagnostic use of tests or even with analytical methods. Making appropriate analytical instruments could have been sufficient. But now that it has entered the clinical field, advising on the relative merits of tests, industry should involve itself much further with the problem; it should invest in training, learn about biological variation and statistics,clinical relevance, parametric testing etc., instead of simply laying out another advertisement each time a new test is discovered and trying to sell it like a new vitamin preparation. And this will require much closer and more systematic cooperation with the profession (the emerging European Committee for Clinical Laboratory Standards, ECCLS, may be the framework for such cooperation). As far as instruments are concerned we would like them to be rugged and safe. Material and reagent should be used at the ultramicro level. Measuring devices should be accurate and stable. Flexibility should result from modularity and miniaturization. Mechanical movements should be quick: much quicker than their manual counterparts. Instruments should stem from a systems analysis of every aspect of laboratory work from and including blood withdrawal to reporting. Every available analytical technique should be envisaged, not just visible absorption spectometry. And additionally all these facets may be automated – why not?

REFERENCES

[1] Analysis 79, 'Automation in industrial and clinical chemistry' Symposium in London, 16-18 July 1979.
[2] International Union of Pure and Applied Chemistry Information Bulletin No 3 1978, 233.
[3] Thesis No 905 F-H Schoff (1979) University Louis Pasteur, France.

